# Supplementary material for: Effectiveness of a Mobile Application for Postpartum Depression Self-Management: Evidence from a Randomised Controlled Trial in South Korea
Source: Healthcare (Basel). 2022 Oct 31;10(11):2185. doi: 10.3390/healthcare10112185 (PMC9690421; doi:10.3390/healthcare10112185)
Supplement: Supplementary file 1 [file healthcare-10-02185-s001.zip › healthcare-1965890-supplementary.pdf]

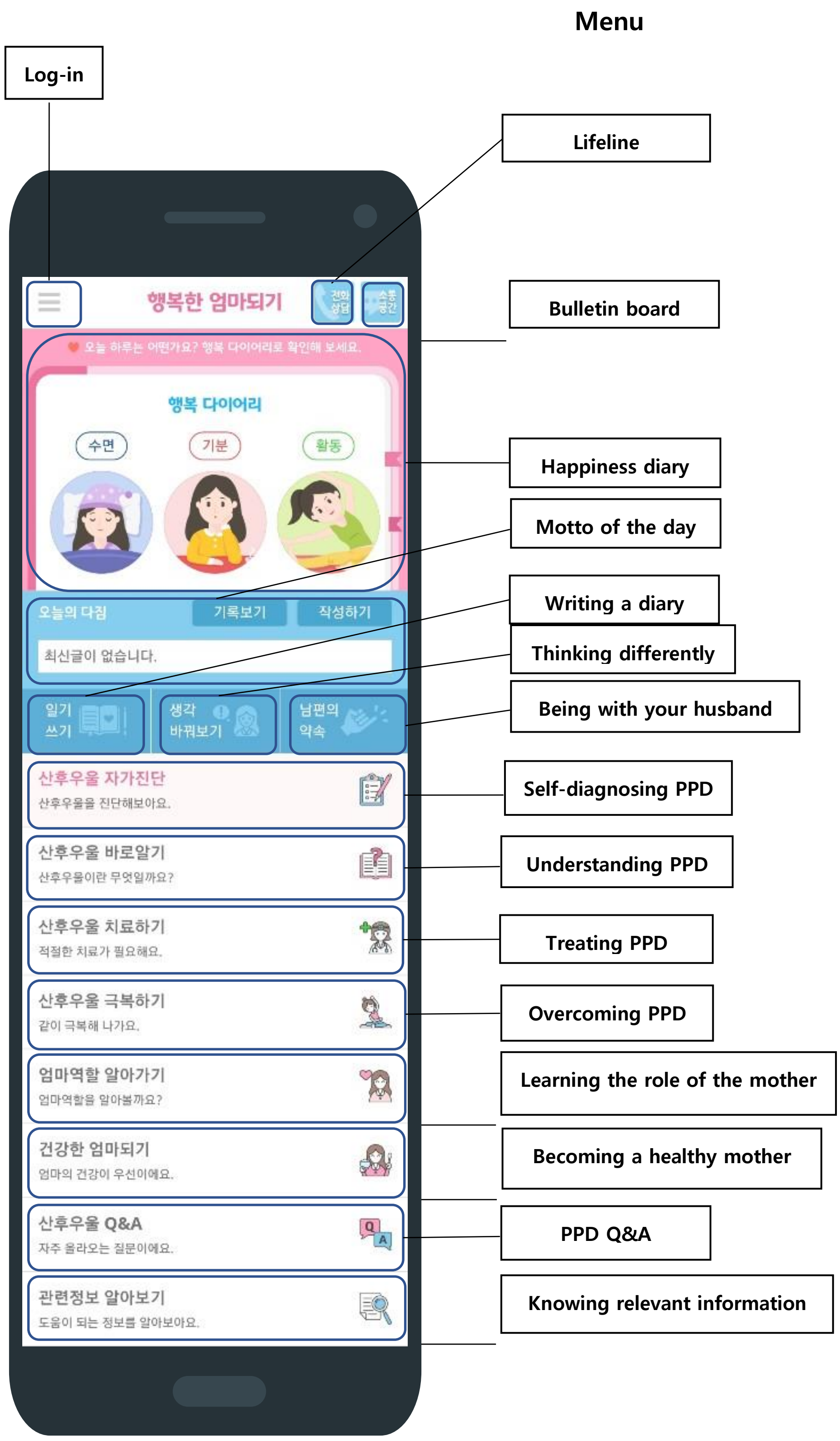

Figure S1. Main screen of the Happy Mother app.

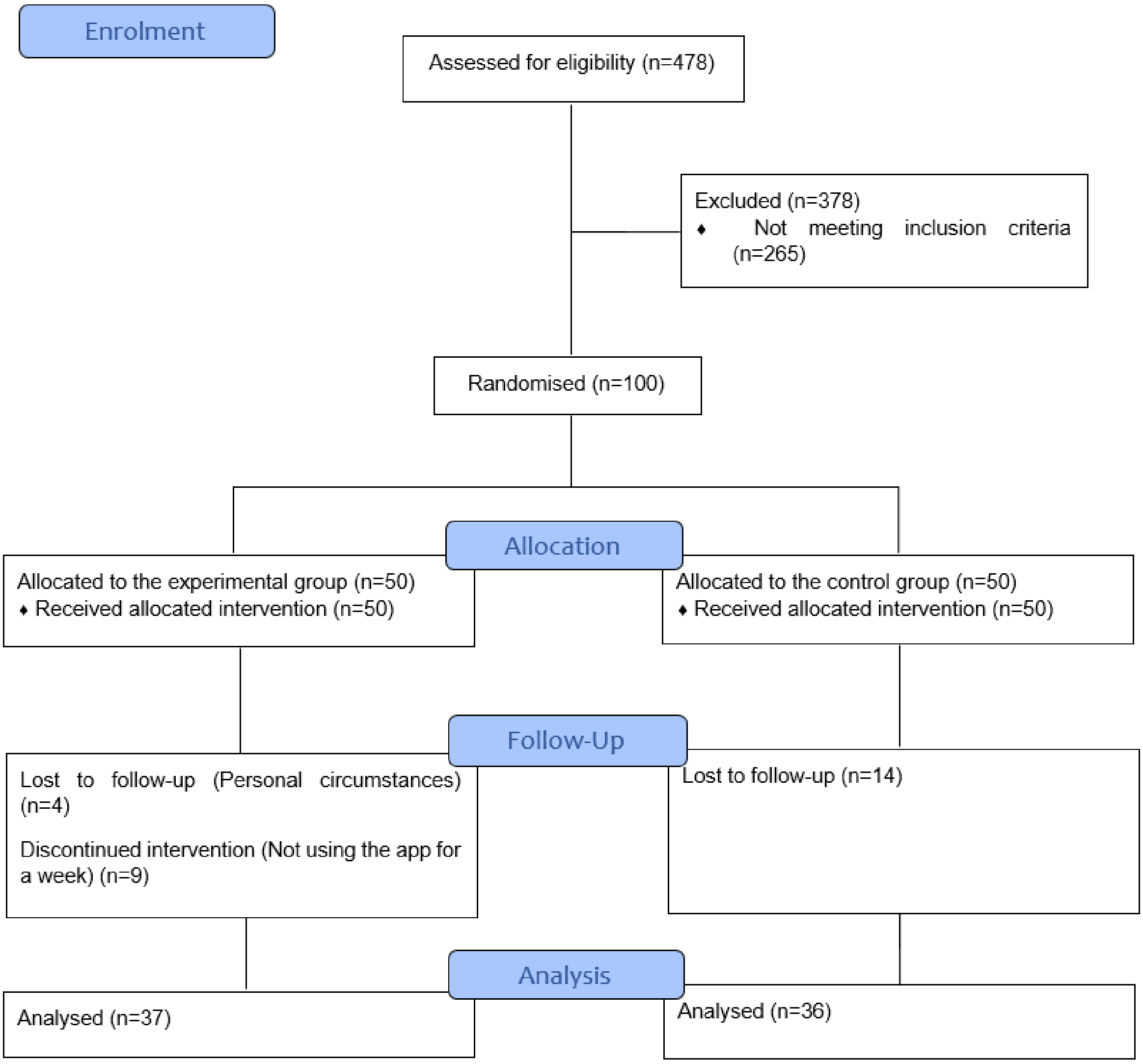

Figure S2. Participant selection flow

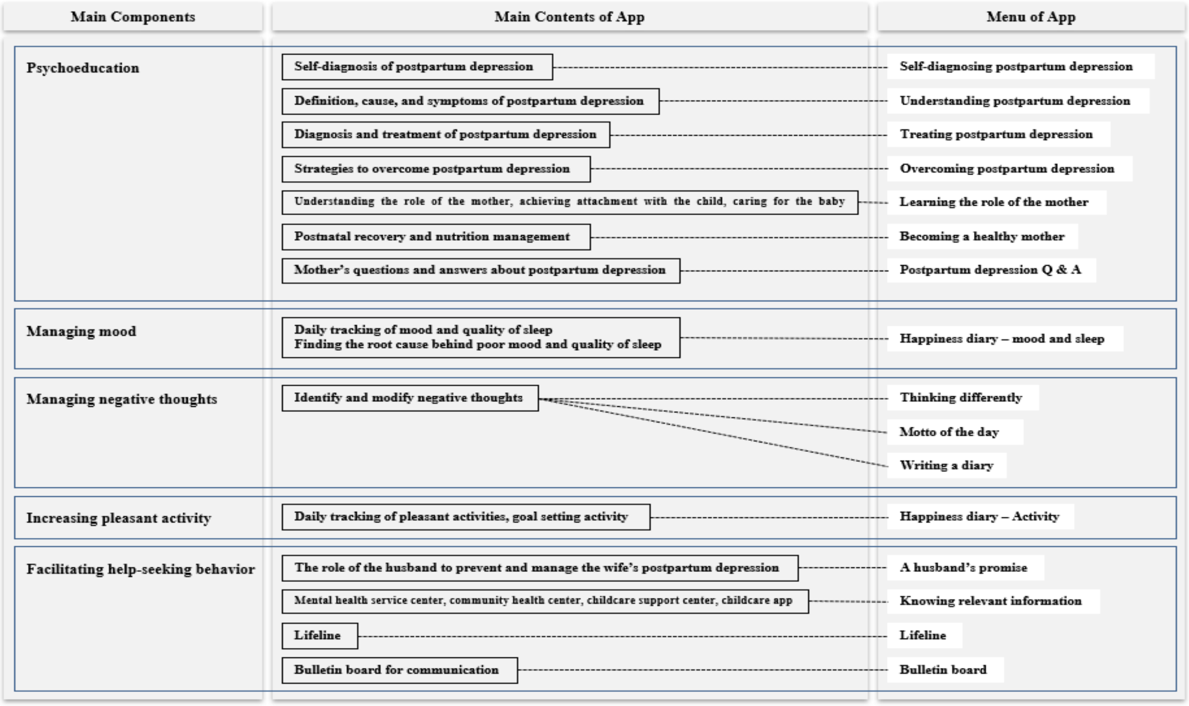

Figure S3. The cognitive behavioural therapy (CBT) themes of the Happy Mother app

CONSORT 2010 checklist of information to include when reporting a randomised trial*

Section/Topic	Item No	Checklist item	Reported on page No
**Title and abstract**			
	1a	Identification as a randomised trial in the title	1
	1b	Structured summary of trial design, methods, results, and conclusions	1
**Introduction**			
Background and objectives	2a	Scientific background and explanation of rationale	2
	2b	Specific objectives or hypotheses	2
**Methods**			
Trial design	3a	Description of trial design (such as parallel, factorial) including allocation ratio	2
	3b	Important changes to methods after trial commencement (such as eligibility criteria), with reasons	N.A
Participants	4a	Eligibility criteria for participants	2
	4b	Settings and locations where the data were collected	2
Interventions	5	The interventions for each group with sufficient details to allow replication, including how and when they were actually administered	2-4
Outcomes	6a	Completely defined pre-specified primary and secondary outcome measures, including how and when they were assessed	4-6
	6b	Any changes to trial outcomes after the trial commenced, with reasons	N.A
Sample size	7a	How sample size was determined	2

	7b	When applicable, explanation of any interim analyses and stopping guidelines	N.A
Randomization:			
Sequence generation	8a	Method used to generate the random allocation sequence	2
	8b	Type of randomization; details of any restriction (such as blocking and block size)	2
Allocation concealment mechanism	9	Mechanism used to implement the random allocation sequence (such as sequentially numbered containers), describing any steps taken to conceal the sequence until interventions were assigned	2-4
Implementation	10	Who generated the random allocation sequence, who enrolled participants, and who assigned participants to interventions	2-4
Blinding	11a	If done, who was blinded after assignment to interventions (for example, participants, care providers, those assessing outcomes) and how	2-4
	11b	If relevant, description of the similarity of interventions	N.A
Statistical methods	12a	Statistical methods used to compare groups for primary and secondary outcomes	6
	12b	Methods for additional analyses, such as subgroup analyses and adjusted analyses	N.A
**Results**			
Participant flow (a diagram is strongly recommended)	13a	For each group, the numbers of participants who were randomly assigned, received intended treatment, and were analysed for the primary outcome	Figure S3
	13b	For each group, losses and exclusions after randomisation, together with reasons	3,4
Recruitment	14a	Dates defining the periods of recruitment and follow-up	2-4
	14b	Why the trial ended or was stopped	N.A
Baseline data	15	A table showing baseline demographic and clinical characteristics for each group	6-8

Numbers analyzed	16	For each group, number of participants (denominator) included in each analysis and whether the analysis was by original assigned groups	2,3
Outcomes and estimation	17a	For each primary and secondary outcome, results for each group, and the estimated effect size and its precision (such as 95% confidence interval)	6-11
	17b	For binary outcomes, presentation of both absolute and relative effect sizes is recommended	N.A
Ancillary analyses	18	Results of any other analyses performed, including subgroup analyses and adjusted analyses, distinguishing pre-specified from exploratory	N.A
Harms	19	All important harms or unintended effects in each group (for specific guidance see CONSORT for harms)	N.A
**Discussion**			
Limitations	20	Trial limitations, addressing sources of potential bias, imprecision, and, if relevant, multiplicity of analyses	13,14
Generalizability	21	Generalizability (external validity, applicability) of the trial findings	12-14
Interpretation	22	Interpretation consistent with results, balancing benefits and harms, and considering other relevant evidence	12-14
**Other information**			
Registration	23	Registration number and name of trial registry	N.A
Protocol	24	Where the full trial protocol can be accessed, if available	N.A
Funding	25	Sources of funding and other support (such as supply of drugs), role of funders	14

*We strongly recommend reading this statement in conjunction with the CONSORT 2010 Explanation and Elaboration for important clarifications on all the items. If relevant, we also recommend reading CONSORT extensions for cluster randomised trials, non-inferiority and equivalence trials, non-pharmacological treatments, herbal interventions, and pragmatic trials. Additional extensions are forthcoming: for those and for up to date references relevant to this checklist, see www.consort-statement.org.

Table S1. CONSORT checklist

1) How the app was helpful

(Theme 1) Self-monitoring and planning life using the Happiness Diary

'It was nice to see the mood, sleep, and activity trends on the graph. It was especially good to be active, but it was better to plan what I was going to do today and to see what I was doing in the evening'. (Mothers 6, 8)

(Theme 2) Functions to help manage depression

'I used "thinking positively". I had always blamed my husband, [but] I learned to think positively and was able to control my mind a little bit'. (Mothers 6, 8)

'The ability to share with my husband was helpful. I felt less lonely than before because my husband supported me'. (Mothers 1, 4, 6)

(Theme 3) Helpful parenting information

'Information about the role of a mother and how to raise a baby was memorable and helpful'. (Mothers 1, 5, 6)

'I actually used to make good use of the Moonlight Children's Hospital or the application for childcare'. (Mothers 5, 6, 7)

(Theme 4) Psychological comfort through the bulletin board

'I left a message on the bulletin board when I was having a hard time, and it comforted me a lot because the manager left a comment'. (Mother 4)

2) Improvements needed

**(Theme 1) Supplement the functions of the Happy Diary**
'Since the quality of sleep was presented only as a numerical value, it was difficult to accurately determine how much my quality of sleep was'. (Mother 1)
**(Theme 2) More training on altering dysfunctional thoughts**
'I did not use the "Thinking Differently" of the app much. I hesitated to use it because it was not a familiar method to me. I think we need more training on thinking differently in real life'. (Mothers 3)
**(Theme 3) Periodical updates**
'The bulletin board wasn't activated, so I didn't have the courage to write'. (Mothers 3, 6-8)<br>*'The app should be updated regularly as I want to get a lot of new information'. (Mothers 4, 8)*

Table S2. Actual statement of the open ended-interviews

Menu	Detailed menu	Whole period		Within 8 weeks		After 9 weeks	
		Total number of uses	Total number of users (%)	Total number of uses	Total number of users (%)	Total number of uses	Total number of users (%)
Happiness diary		4073	37(100%)	2067	37(100%)	2006	36(97.30%)
	Mood	1317	37(100%)	673	37(100%)	644	35(94.59%)
	Sleep	1255	37(100%)	628	37(100%)	627	36(97.30%)
	Activity	1501	37(100%)	766	37(100%)	735	35(94.59%)
Self-diagnosing PPD		135	37(100%)	99	37(100%)	36	16(43.24%)
Understanding PPD		51	17(45.95%)	43	14(37.84%)	8	3(8.11%)
Treating PPD		79	19(51.35%)	63	16(43.24%)	16	5(13.51%)
Overcoming PPD		652	37(100%)	583	37(100%)	150	31(83.78%)
	Thinking positively	83	16(43.24%)	65	16(43.24%)	18	5(13.51%)
	Being with your husband	35	9(24.32%)	26	9(24.32%)	9	4(10.81%)
Learning the role of the mother		231	31(83.78%)	128	31(83.78%)	22	9(24.32%)
Becoming a healthy mother		112	23(62.16%)	99	21(56.76%)	13	6(16.22%)
PPD Q & A		72	36(97.30%)	70	35(94.59%)	2	2(5.41%)
Knowing relevant information		198	36(97.30%)	189	36(97.30%)	9	4(10.81%)
Bulletin board		220	36(97.30%)	143	36(97.30%)	77	14(37.84%)
	(Participants actually created)	14	7(18.92%)	10	5(13.51%)	4	4(10.81%)
Lifeline		61	36(97.30%)	50	36(97.30%)	11	9(24.32%)

Motto of the day	238	35(94.59%)	174	33(89.19%)	64	17(45.95%)
Writing a diary	153	36(97.30%)	111	35(94.59%)	42	17(45.95%)

Table S3. Percentage of experimental groups using the app (N=37)
